# Evaluation of patient- versus provider-collected vaginal swabs for microbiome analysis during pregnancy

**DOI:** 10.1186/s13104-018-3809-4

**Published:** 2018-10-05

**Authors:** Kristine M. Wylie, Stephanie A. Blankenship, Methodius G. Tuuli, George A. Macones, Molly J. Stout

**Affiliations:** 1 Department of Pediatrics, Washington University School in St. Louis School of Medicine, St. Louis, MO USA; 2 The McDonnell Genome Institute, Washington University in St. Louis School of Medicine, St. Louis, MO USA; 3 Department of Obstetrics and Gynecology, Washington University in St. Louis School of Medicine, 660 S. Euclid, Box 8064, St. Louis, MO 63110 USA

**Keywords:** 16S rRNA, Patient-collected sample, Pregnancy, Vaginal microbiome

## Abstract

**Objective:**

We aimed to evaluate if patient- and provider-collected vaginal swabs in pregnant women reflect similar bacterial community characteristics. Pregnant patients performed a self-collected vaginal swab, then underwent a provider-collected swab via speculum exam. DNA pyrosequencing of the 16S rRNA gene V1V3 and V3V5 variable regions was performed. Relative abundance of taxa, alpha diversity, and beta diversity of patient- and provider-collected swabs were compared.

**Results:**

Ninety-four vaginal swabs from 47 women were analyzed. On non-metric multi-dimensional scaling plots, paired patient- and provider-collected swabs clustered closely. The median Pearson correlation coefficient was 0.993 (interquartile range 0.951–0.999) for V1V3 and 0.987 (interquartile range 0.902–0.999) for V3V5. Among paired V1V3 and V3V5 sequences, 83.0% and 73.9% showed strong Pearson correlation (> 0.9), respectively, between patient- and provider-collected swabs; V1V3 and V3V5 sequences with weaker Pearson correlation (< 0.9) had correlation coefficients 0.57–0.89 and 0.49–0.89, respectively. No taxa were preferentially detected by sampling method, with relative abundance of taxa highly conserved. No significant difference in Shannon diversity for V1V3 (p = 0.22) and V3V5 (p = 0.11) sequences among paired samples was seen. We demonstrate that bacterial communities defined from patient- and provider-collected vaginal swabs in pregnant women are similar, validating utilization of patient-collected swabs for vaginal bacterial microbiome sampling during pregnancy.

## Introduction

In recent years, culture-independent, phylogenetic characterization of microbial communities using 16S ribosomal RNA (16S rRNA) gene sequences directly extracted from biological samples has provided a broad understanding of vaginal microbial communities among reproductive age women [1]. Important work by Aagaard et al. demonstrated that the vaginal microbiome of pregnancy uniquely differs compared to the non-pregnant state. More specifically, the vaginal communities of pregnant women are characterized by lower richness and diversity, but higher stability than those of non-pregnant women [2].

Additionally, new data suggests that alterations of the vaginal microbiota may be associated with adverse obstetric outcomes such as preterm birth [3, 4]. Examination of community membership, behavior, and community dynamics of the vaginal microbiome over pregnancy may further elucidate mechanisms that predispose women to preterm birth. However, these studies are dependent on recruitment of pregnant women and optimization of experimental processes to minimize patient burden while preserving the opportunity for reliable and scientifically informative results.

Women find it easier and in most cases prefer self-collection over collection of vaginal samples by clinicians via speculum exam [5–7]. Studies performed using patient-collected swabs demonstrate adequate diagnostic accuracy for clinical infections such as gonorrhea and Chlamydia [7]. Microbiome analysis in a non-pregnant cohort also showed similar vaginal communities in patient- and provider-based sampling methods [8]. However, data are not currently available regarding the representation of microbial species composition in patient-collected swabs compared to provider-collected swabs among pregnant women. Considering the maternal physiologic changes in pregnancy, including the gravid abdomen and increased physiologic vaginal discharge, we aimed to evaluate if patient-collected swabs of the mid-vagina in pregnancy reflect the same microbial community characteristics as provider-collected swabs obtained during speculum exam. Demonstrating similarity in patient- versus provider-collection during pregnancy may have wide ranging applicability from research protocols to clinical diagnostic testing.

## Main text

### Methods

We performed a cross-sectional study of women within our prospective cohort examining vaginal microbiome trends over pregnancy [3]. The Washington University in St. Louis Human Research Protection Office approved this study and informed consent was obtained from all study participants. Inclusion criteria for this analysis were willingness to undergo speculum examination, willingness to perform self-sampling, and singleton gestation. Exclusion criteria were cervical cerclage and vaginal or intramuscular progesterone therapy. The patient was asked to self-collect a swab immediately prior to their provider visit at the same time that their urine specimen for routine obstetric care was obtained. The patient was provided a sterile dual-tipped rayon swab with instructions to remove the swab from the tube, spread her labia with her non-dominant hand, and then using her dominant hand, insert the swab approximately 3 cm into the vaginal canal, twist the swab within the vaginal canal to wipe in several full circles within the vagina, and remove the swab to place it back into the sterile collection tube. After the self-collection was complete the patient would see her provider and undergo a speculum examination for a provider-collected swab. For provider-collected swabs, a sterile speculum was introduced into the vagina, and the swab was applied to both mid-vaginal lateral sidewalls 3–5 times per side and placed back into the sterile collection tube. Swabs were immediately stored at − 20 °C until transportation to the laboratory where they were frozen at − 80 °C until DNA extraction.

DNA sequencing and analysis were performed according to the protocols used by the Human Microbiome Project [9–11]. In brief, DNA was isolated using the manufacturer’s protocol from the MoBio PowerSoil DNA Isolation Kit (MO BIO Laboratories, Carlsbad CA). Extracted DNA samples were stored in solution at − 80 °C until sequencing. Pyrosequencing of V1V3 and V3V5 variable regions of the 16S rRNA gene was carried out on the Roche 454 GS FLX.

Sequences were binned by samples using sample-specific barcodes, allowing one mismatch. Sequences were removed from subsequent analysis if the average quality score was less than 35, the read length was less than 200 bases, or the sequences was classified as chimeric by ChimeraSlayer [12]. One sample contained < 1000 reads in the V3V5 variable region, so that sample and its pair were removed from further analysis. Sequences that passed quality filters were classified using the Ribosomal Database Project naïve bayesian classifier, version 2.5 with training set 10 [13]. Each sample was subsampled to the lowest number of read counts among samples in the data sets (1753 for V1V3 and 2135 for V3V5).

Shannon diversity was compared between paired patient- and provider-collected samples using the Wilcoxon signed rank test. Beta diversity (Bray–Curtis dissimilarity) was calculated and visualized using non-metric multi-dimensional scaling (NMDS) plots [14, 15]. The Pearson correlation coefficient was calculated for the taxa detected in the pair of patient-collected and speculum-collected swabs from each individual. We generated stacked bar charts to illustrate the taxonomic composition of the samples [16]. Taxa with less than five reads detected were removed, and the resulting data set was used to carry out linear discriminant analysis effect size (LEfSe) to determine whether there were taxa that were preferentially detected in the patient-collected or provider-collected samples [17].

### Results

We obtained 94 vaginal swabs from 47 women. An average of 5572 16S rRNA gene sequences were obtained per sample. Median gestational age of sampling was 20.1 weeks (interquartile range 12.4–28.0 weeks) and ranged from 5 to 33 weeks. Our patient cohort was predominantly black (74.5%) and obese with BMI > 30 (73%). Median BMI was 29.2 with interquartile range of 25.6–35.8. In addition, 21.7% (n = 10 women) in our cohort delivered preterm.

**Fig. 1:**
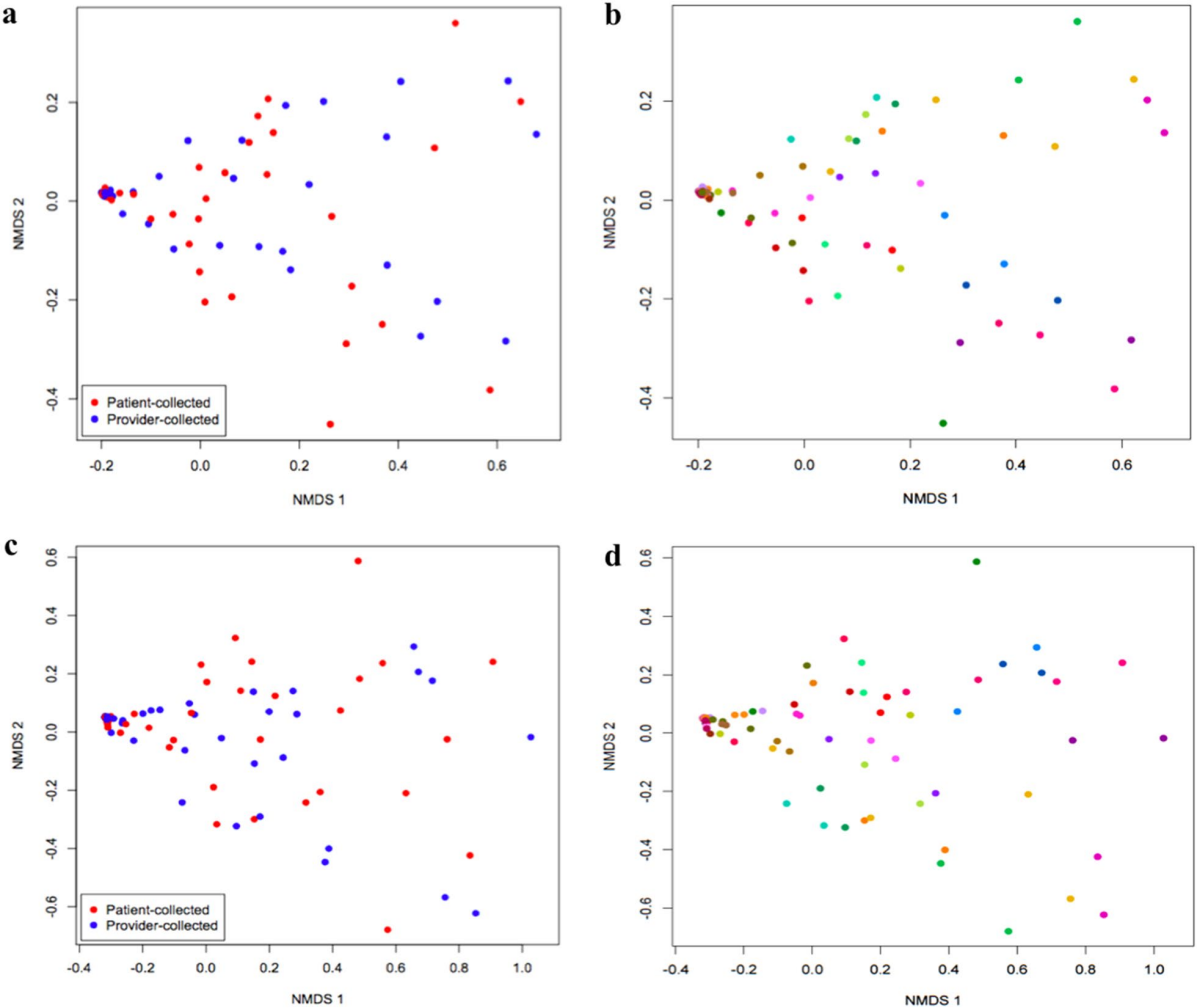
NMDS plots for V1V3 and V3V5 regions. **a** NMDS plot for V1V3 regions for patient- (red) versus provider-collected (blue) vaginal swabs. **b** NMDS plot for V1V3 regions for patient- and provider-collected vaginal swabs, with the same color representing the same patient. **c** NMDS plot for V3V5 regions for patient- (red) versus provider-collected (blue) vaginal swabs. **d** NMDS plot for V3V5 regions for patient- and provider-collected vaginal swabs, with the same color representing the same patient

Alpha diversity measures demonstrated similarity between patient- and provider-based collection methods. There was no difference seen in Shannon diversity between patient- versus provider-collected swabs in either V1V3 (p = 0.22) or V3V5 (p = 0.11) sequences. NMDS plots were created to evaluate the similarity of bacterial communities between patient-collected and provider-collected swabs. Patient-collected swabs did not cluster separately from provider-collected swabs for both the V1V3 and V3V5 regions (Fig. 1a and c, respectively). Paired patient- and provider-collected swabs sampled from the same patient at the same time point clustered closely for both the V1V3 and V3V5 regions (Fig. 1b and d, respectively).

Pearson correlation coefficients were then used to quantify similarity in bacterial communities of patient- versus provider-collected swabs collected from the same patient at the same time. Among paired samples of 16S rRNA sequences from V1V3, 39 of 47 paired samples (83.0%) showed strong correlation between provider- and patient-collected swabs (Pearson correlation > 0.9). Additionally, 34 of 46 paired samples (73.9%) of 16S rRNA sequences from V3V5 also demonstrated strong correlation (Pearson correlation > 0.9).

**Fig. 2:**
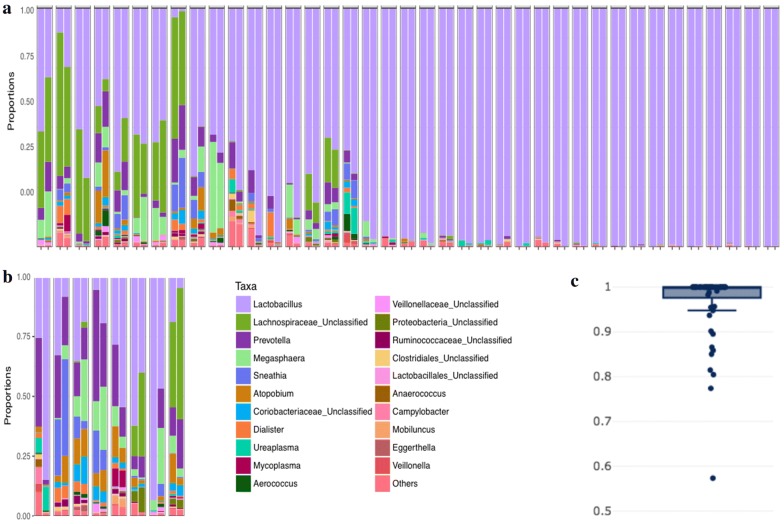
Pearson correlation for V1V3 regions. **a** Relative abundance of taxa in V1V3 regions found among paired patient- (left) and provider-collected (right) swabs from the same patient with Pearson correlation coefficient > 0.9, ordered from least strongly to most strongly correlated. **b** Relative abundance of taxa in V1V3 regions found among paired patient- (left) and provider-collected (right) swabs from the same patient with Pearson correlation coefficient < 0.9, ordered from least strongly to most strongly correlated. **c** Box plot demonstrating Pearson correlation coefficients for correlated microbial communities defined for V1V3 regions in paired patient- and provider-collected swabs from the same patient

**Fig. 3:**
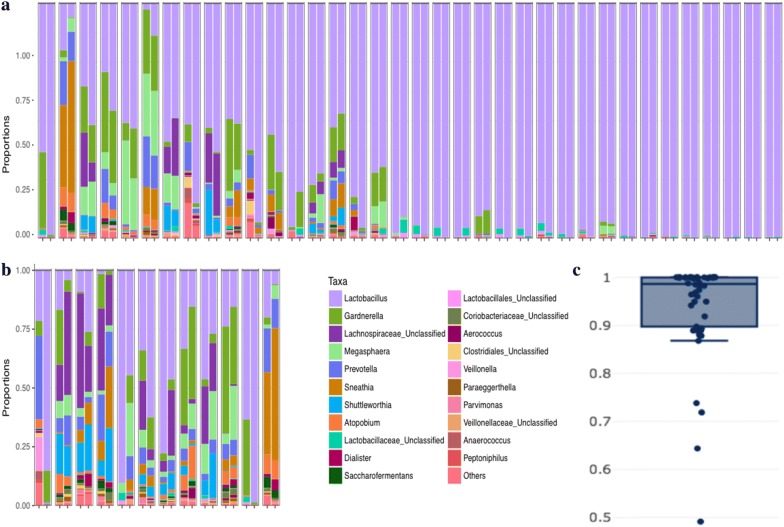
Pearson correlation for V3V5 regions. **a** Relative abundance of taxa in V3V5 regions found among paired patient- (left) and provider-collected (right) swabs from the same patient with Pearson correlation coefficient > 0.9, ordered from least strongly to most strongly correlated. **b** Relative abundance of taxa in V3V5 regions found among paired patient- (left) and provider-collected swabs (right) from the same patient with Pearson correlation coefficient < 0.9, ordered from least strongly to most strongly correlated. **c** Box plot demonstrating Pearson correlation coefficients for correlated microbial communities defined for V3V5 regions in paired patient- and provider-collected swabs from the same patient

The relative distribution of taxa found among V1V3 regions in provider- and patient-collected samples with strong correlation is shown in Fig. 2a, along with 8 samples in the V1V3 region with weaker correlation shown in Fig. 2b (Pearson correlation < 0.9). The V1V3 sequences with Pearson correlation < 0.9 had correlation coefficients ranging from 0.57 to 0.89. The median Pearson correlation coefficient for V1V3 sequences overall was 0.993 (interquartile range 0.951–0.999) as shown in Fig. 2c. Similarly, the distribution of taxa found among V3V5 sequences in patient- and provider-collected samples with strong correlation is shown in Fig. 3a, along with 12 samples in the V3V5 region with weaker correlation shown in Fig. 3b (Pearson correlation < 0.9) with correlation coefficients ranging from 0.49 to 0.89. The median Pearson correlation coefficient for V3V5 sequences overall was 0.987 (interquartile range 0.902–0.999), seen in Fig. 3c. V1V3 and V3V5 samples with weaker correlation between provider- and patient-collected swabs differed in relative abundances of different microbial taxa in each sample type. However, the taxa detected were highly conserved between paired samples. There were no taxa that were preferentially detected by self-sampling or provider-sampling.

### Discussion

The present study demonstrates that patient-collected vaginal swabs reflect highly similar bacterial communities as provider-collected specimens in pregnant women. In both the V1V3 and V3V5 regions, the patient-collected swabs did not cluster separately from provider-collected swabs. Additionally, paired patient- and provider-collected swabs sampled from the same patient at the same time point clustered closely, suggesting that bacterial microbial communities identified by patient self-sampling are similar to those obtained from provider-based sampling. When differences in patient versus provider collection are present they generally appear to be due to changes in relative abundances of taxa present, but not due to specific taxa that are inherently different by sampling strategy. Even with the variations in relative abundances, overall differences in alpha diversity did not differ between patient and provider collections.

Previous studies have similarly confirmed patient-collected swabs in non-pregnant women have similar detection rates and diagnostic accuracy for identifying bacterial vaginosis, *Candida vaginalis*, *Neisseria gonorrhea*, *Chlamydia trachomatis*, group B streptococcus and human papillomavirus as provider-collected swabs obtained during speculum exam [5–7, 18–22]. Forney et al. also revealed patient-collected vaginal samples and provider-collected swabs of the mid-vagina in non-pregnant women had the same microbial diversity based on community taxa composition and relative abundance [8]. Our study validates the use of patient-collected swabs for the detection of vaginal bacterial community composition during pregnancy.

Vaginal bacterial communities have the potential to differ according to sampling method in pregnant women as a result of various factors. Both sampling site and sampling method have previously been shown to influence the composition of vaginal microbial communities within individuals [2, 23]. In the case of patient-collected vaginal samples it is plausible that taxa might be missed, or may be more or less abundant than expected, by not controlling the exact vaginal location where the sampling occurred. Our patients were sampled at a wide range of gestational ages, predominantly in the late second and early third trimesters. Additionally, our patient population is predominantly obese with BMI > 30. Thus, reaching over the gravid abdomen to sample the inner vagina may contribute to sampling bias, as they may not be able to sufficiently reach the mid-vagina. Furthermore, sequential, repeated sampling of the vagina by the patient followed by the provider could alter microbial populations. Nonetheless, our data suggest that patient collection and provider collection produce similar results for vaginal community characteristics with high correlation between sample types, no differences in detection of specific taxa by sample type, and no systematic differences in higher or lower alpha diversity measurement by sample type.

## Limitations

Our study has potential limitations. While these findings suggest that sampling methodology does not result in systematic differences in vaginal microbiome community characteristics in pregnant women, we observed subtle differences between patient- and provider-collected swabs. These may be due to minor differences attributable to the sample collection methods *or*, alternatively, are due to the inherent variability between two serial biologic samplings (i.e. would have been as likely to be present with provider speculum exam as with two serial swabs obtained). Additionally, this study assesses only bacterial components of the microbiome and no conclusions can be made about the similarity of viral or fungal components of the microbiome by sampling method. Lastly, our study only evaluated sample collection methods in asymptomatic pregnant women to assess vaginal microbiome composition; however, it remains uncertain whether patient- and provider-based sampling would be equally effective for diagnostic point-of-care testing and evaluation of clinical infections in pregnant women, as has been previously demonstrated in non-pregnant women [5–7, 18–22]. Future interventional studies are required to test clinical applications and cost-effectiveness.

### Conclusions

Use of patient-collected samples during pregnancy may have wide applicability. The vaginal microbiome is dynamic and characterizing these fluctuations may be of biological and clinical significance [24, 25]. Use of patient-collected samples would allow closely-spaced longitudinal sampling and increase the ability to detect subtle temporal changes in the vaginal microbiome over gestation and postpartum. Patient self-collection could improve cost- and time-efficiency for both patients and providers. Overall, these data demonstrate that the vaginal bacterial communities are similar in patient- and provider-collected swabs in pregnancy. Our findings suggest that utilizing less invasive patient self-collection is an acceptable sampling method to study vaginal microbiome dynamics during pregnancy.

## Abbreviations

16S rRNA: 16S ribosomal RNA
NMDS: non-metric multi-dimensional scaling
LEfSe: linear discriminant analysis effect size
